# Superior suppression of serum estrogens during neoadjuvant breast cancer treatment with letrozole compared to exemestane

**DOI:** 10.1007/s10549-024-07313-x

**Published:** 2024-04-23

**Authors:** Bjørn-Erik Bertelsen, Bjørg Almås, Kamilla Fjermeros, Kristin Viste, Stephanie Beate Geisler, Torill Sauer, Knut Selsås, Jürgen Geisler

**Affiliations:** 1 Hormone Laboratory, Department of Medical Biochemistry and Pharmacology, Haukeland, University Hospital, Bergen, Norway; 2 Department of Oncology, Akershus University Hospital, Lørenskog, Norway; 3 Department of Pathology, Akershus University Hospital, Lørenskog, Norway; 4 Institute of Clinical Medicine, Faculty of Medicine, University of Oslo, Oslo, Norway; 5 Department of Breast- and Endocrine Surgery, Akershus University Hospital, Lørenskog, Norway

**Keywords:** Breast cancer, Neoadjuvant, Aromatase, Letrozole, Exemestane, Estrogens

## Abstract

**Purpose:**

The aromatase inhibitor letrozole and the aromatase inactivator exemestane are two of the most pivotal cancer drugs used for endocrine treatment of ER-positive breast cancer in all phases of the disease. Although both drugs inhibit CYP19 (aromatase) and have been used for decades, a direct head-to-head, intra-patient-cross-over comparison of their ability to decrease estrogen synthesis in vivo is still lacking.

**Methods:**

Postmenopausal breast cancer patients suitable for neoadjuvant endocrine therapy were randomized to receive either letrozole (2.5 mg o.d.) or exemestane (25 mg o.d.) for an initial treatment period, followed by a second treatment period on the alternative drug (intra-patient cross-over study design). Serum levels of estrone (E1), estradiol (E2), letrozole, exemestane, and 17-hydroxyexemestane were quantified simultaneously using a novel, ultrasensitive LC–MS/MS method established in our laboratory.

**Results:**

Complete sets of serum samples (baseline and during treatment with letrozole or exemestane) were available from 79 patients, including 40 patients starting with letrozole (cohort 1) and 39 with exemestane (cohort 2). Mean serum estrone and estradiol levels in cohort 1 were 174 pmol/L and 46.4 pmol/L at baseline, respectively. Treatment with letrozole suppressed serum E1 and E2 to a mean value of 0.2 pmol/L and 0.4 pmol/L (*P* < 0.001). After the cross-over to exemestane, mean serum levels of E1 and E2 increased to 1.4 pmol/L and 0.7 pmol/L, respectively. In cohort 2, baseline mean serum levels of E1 and E2 were 159 and 32.5 pmol/L, respectively. Treatment with exemestane decreased these values to 1.8 pmol/L for E1 and 0.6 pmol/L for E2 (*P* < 0.001). Following cross-over to letrozole, mean serum levels of E1 and E2 were significantly further reduced to 0.1 pmol/L and 0.4 pmol/L, respectively. Serum drug levels were monitored in all patients throughout the entire treatment and confirmed adherence to the protocol and drug concentrations within the therapeutic range for all patients. Additionally, Ki-67 values decreased significantly during treatment with both aromatase inhibitors, showing a trend toward a stronger suppression in obese women.

**Conclusion:**

To the best of our knowledge, we present here for the first time a comprehensive and direct head-to-head, intra-patient-cross-over comparison of the aromatase inhibitor letrozole and the aromatase inactivator exemestane concerning their ability to suppress serum estrogen levels in vivo. All in all, our results clearly demonstrate that letrozole therapy results in a more profound suppression of serum E1 and E2 levels compared to exemestane.

## Introduction

Antihormonal therapy is one of the mainstays in the treatment of estrogen receptor (ER) positive breast cancer, comprising 70–80% of all cases. During the last two decades, aromatase inhibitors (AIs) of the “third generation” pushed aside earlier generations and have been the first choice as antihormonal drugs in postmenopausal women [1–3]. Following several decades with aromatase inhibitors given as monotherapy, aromatase inhibitors are now the primary antihormonal backbone for treatment of ER-pos. breast cancer in all phases of the disease, including novel treatment combinations like AIs given in concert with CDK4,6 inhibitors [4]. While letrozole has been widely used as a third-generation, non-steroidal aromatase inhibitor, exemestane is the most important steroidal aromatase inactivator globally. Both drugs have been extensively investigated concerning their ability to suppress both serum and tissue estrogens by our group and others [5–11]. The major question whether the observed estrogen suppression is correlated to the clinical outcome has been addressed and the principal understanding is that a more complete estrogen suppression is explaining the superiority of the third-generation drugs compared to the earlier generations of AIs [1]. Notably, Ingle et al. recently confirmed a link between suboptimal estrogen suppression during adjuvant therapy with anastrozole, another third-generation non-steroidal AI, and an increased risk of an early breast cancer event [12]. This underscores the importance of the pharmacological potency of a particular drug and patient adherence.

To the best of our knowledge, no direct head-to-head, intra-patient-cross-over comparison of the estrogen suppression caused during treatment with these two pivotal breast cancer drugs, letrozole and exemestane, has been performed. This is primarily due to the absence of suitable clinical trials and lack of analytical methods capable to detect estrogen levels in the ultra-low concentrations during ongoing therapy with comparably high daily doses (25 mg × 1) of the steroidal compound exemestane. However, it has to be mentioned that Robarge et al. published results of a randomized, multicenter trial of postmenopausal women with early breast cancer treated with either letrozole (*n* = 241) or exemestane (*n* = 228) and concluded that letrozole caused a greater suppression of plasma E1 and E1S than exemestane [9].

In this report, we present the results of a phase II neoadjuvant trial, performed at the Akershus University Hospital in the Oslo area, Norway. Postmenopausal women with locally advanced or large T2 tumors, strongly ER-pos. breast cancer were selected for presurgical therapy with either letrozole or exemestane upfront followed by a second treatment period on the alternative drug. Serum estrogen and drug levels were measured using a state-of-the-art liquid chromatography tandem mass spectrometry (LC–MS/MS) method, which has been previously published in detail [13].

In addition to the obvious major endpoint of this investigation, the suppression of serum estrogen levels, the current study contributes to another important aspect of endocrine therapy in breast cancer patients. Interestingly, several investigators have observed and reported only a partial cross-resistance between non-steroidal AIs such as letrozole and the steroidal compound exemestane, opening for a sequential use of these drugs in the metastatic setting [14, 15]. In fact, this sequence is now established in many recommended breast cancer treatment algorithms around the globe. However, based on preliminary findings indicating that exemestane might be a somewhat weaker aromatase inhibitor compared to letrozole, the clinical observation of responses to exemestane after progression on non-steroidal AIs is hard to explain by traditional concepts of endocrine therapy. Following years of assumptions and uncertainty due to the lack of adequate methods and biobanks, the present manuscript finally enables us to position these two important cancer drugs concerning their potencies as AIs in vivo.

## Methods

### Study design and population

Postmenopausal women with ER-positive, locally advanced breast cancer (cT3-cT4 and/or cN2/N3), suitable for neoadjuvant antihormonal therapy were evaluated for inclusion. In addition, patients with large ER-pos. T2- tumors were also suitable candidates. Postmenopausal status was defined as age above 55 years or age above 50 years and at least 2 years of amenorrhea in addition to LH-, FSH-, and plasma estradiol levels in the postmenopausal range. Limited, non-life-threatening distant metastasis suitable for systemic antihormonal therapy was allowed. In addition, patients with non-clarifiable lesions identified during standard staging procedures, like small micronoduli in the lungs or minor, unclear lesions in the skeleton, often not suitable for further clarification by biopsy, were allowed to participate as progression-free survival was not an endpoint of the substudy presented in the following. All patients were treated at the department of oncology at the Akershus University Hospital, Norway and gave their written informed consent prior to participation. The protocol was approved by the regional ethical committee responsible for the South-East of Norway (reference no. 2015/84). Patients’ characteristics are summarized in detail in Table 1.

**Table 1:** Patient characteristics (NEOLETEXE-trial)

	*n*	%
*n (total)*	102	100
Age (mean)	76	
Age (range)	57–89	
*cT-status*		
cT2	7	6.9
cT3	33	32.4
cT4	62	60.8
*cN-status*		
cN0	63	61.8
cN1	26	25.5
cN2	6	5.9
cN3	7	6.9
MBC	14	13.7
*Histology*		
ER-pos. (> 10%)	102	100
ER-pos. (> 50%)	100	98
ER-pos. (100%)	62	60.8
PGR pos. (> 10%)	77	75.5
PGR pos. (> 50%)	60	58.8
PGR pos. (100%)	17	16.7
HER-2 pos^a^	0	0
TNBC	0	0
*BC-subtypes*		
NST	76	74.5
ILC	21	20.6
MUC	4	3.9
SNEC	1	1

*NST* invasive breast cancer of no special type, *ILC* invasive lobular carcinoma, *MUC* mucinous (colloid) carcinoma, *SNEC* small cell neuroendocrine carcinoma

^a^HER-2 pos. (defined as IHC 3 + or IHC2 + and amplified

**Fig. 1:**
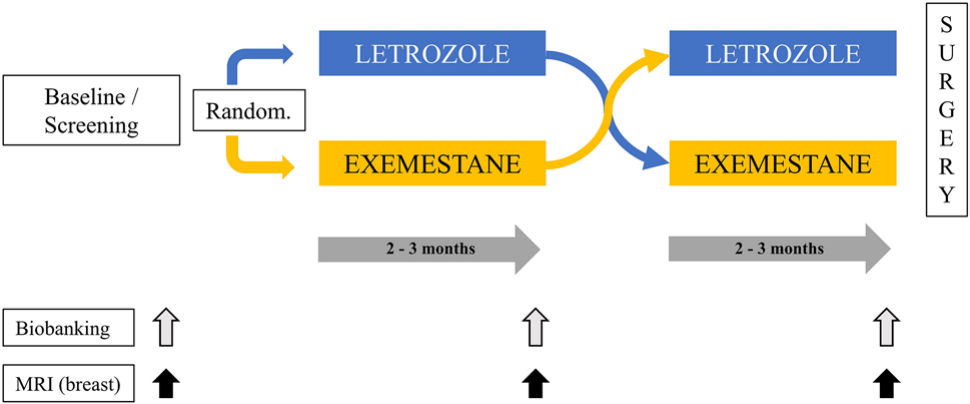
Schematic overview of The NEOLETEXE-trial—neoadjuvant, treatment with letrozole and exemestane in a randomized sequence

The concept of the NEOLETEXE-trial has been published previously [16]. Briefly, all enrolled patients were randomized to either letrozole therapy (2.5 mg once daily p.o.) or exemestane therapy (25 mg once daily p.o.) as initial therapy for at least 2 months to allow a stable state of the endocrine environment. In general, each treatment was given for 3 months (± 1 week), depending on clinically availability of biopsy procedures, etc. Following planned evaluations according to protocol after the first 3 months on treatment (tumor biopsy, blood samples, MRI of the breast, etc.), all patients were crossed over to the alternative therapy for at least another 3 months of systemic therapy. The design of the trial, treatment arms, and the intra-patient cross-over design are briefly summarized in Fig. 1. Finally, after ca. 6 months of neoadjuvant therapy, all patients were evaluated for surgery following the established rules for patients with locally advanced breast cancer. Patients with limited distant metastasis from the time point of diagnosis (*n* = 14) underwent all biopsies and study procedures according to protocol, while the final decision to perform breast surgery was made on an individual basis, as usual in pragmatic trials. For the majority of patients, lacking signs for distant metastasis, adjuvant therapy started directly after surgery including radiotherapy, chemotherapy, endocrine therapy, and bisphosphonates according to the established national guidelines.

### Procedures and measurements

#### Biobanking

Blood samples were drawn at baseline (prior to any cancer therapy), after at least 2 months on the first AI (while still on the drug) and finally after at least 2 months of therapy with the alternative AI. Blood samples included heparin plasma, EDTA plasma, serum, citrate plasma, and EDTA full blood. All samples were obtained by trained study nurses and processed immediately prior to storage at − 80 °C until final analysis. Alongside blood collection, breast tumor biopsies were obtained either by open surgical biopsies or ultrasound-guided vacuum needle biopsies using the Becton Dickinson Elevation Probe (14G) provided by BD (Becton, Dickinson and Company) 1 Becton Drive, Franklin Lakes, NJ 07417, USA.

#### Pathological evaluations

All tumor biopsies were evaluated by highly experienced specialists in breast cancer pathology. The contents of estrogen receptor (ER), progesterone receptor (PGR), human epithelial receptor 2 (HER-2), and tumor grade were described as usual. Only samples with sufficient tumor cell content were used for analysis. In addition, Ki-67 values were provided by the department of pathology at Akershus University Hospital following standard procedures (immunohistochemistry).

#### Measurement of serum estrogens and serum levels of aromatase inhibitors

Concentrations of estradiol, estrone, letrozole, exemestane, and 17-hydroxy-exemestane were quantified simultaneously using an ultrasensitive LC–MS/MS method that has been previously described [13]. Briefly, isotope-labeled internal standards were added to patient samples, calibrators, and quality controls in a 96-deep-well plate. Extraction was performed using hexane:methyl tert-butyl ether and hexane:isopropyl. All steps in the sample preparation were fully automated using a Hamilton Star robot. Extracts were analyzed by LC–MS/MS. The LLOQs for E2 and E1 were 0.8 pmol/L and 0.2 pmol/L, respectively. Results below LLOQ were assigned a value using the formula: LLOQ/2. In employing LLOQ/2 for our calculations, we adhered to an accepted convention in this field, providing a simple method for incorporating left-censored data into our analysis. There are several statistical methods for handling such data, each with its merits. We selected LLOQ/2 for its simplicity, clarity, and the negligible impact of different methods on our case's conclusions. We and others have used the same approach in other published studies [17–19].

## Results

### Patient characteristics

The NEOLETEXE-trial enrolled all in all 102 postmenopausal patients suffering from locally advanced breast cancer. In the present pre-planned subprotocol, we were able to analyze serum samples obtained from 79 individual patients using our novel LC–MS/MS method as previously described [13]. Only patients with serum samples available from all three planned time points (baseline, end of first AI therapy, and end of second AI therapy) were used. Patients with only one or two serum samples were excluded. Patients’ characteristics are summarized briefly in Table 1. The majority of patients in our trial (*n* = 78) were Caucasians. Only 1 patient was born in the People’s Republic of China and lived in Norway when diagnosed with breast cancer. The first cohort, consisting of 40 patients, started with letrozole as the initial therapy and later switched to exemestane. The second cohort, comprising 39 patients, began with exemestane upfront and transitioned to letrozole for the second treatment period. Both AI therapies were given for precisely 3 months, resulting in a total neoadjuvant treatment period of 6 months prior to surgery (± 7 days were allowed for practical reasons).

### Serum estrogen measurements

**Fig. 2:**
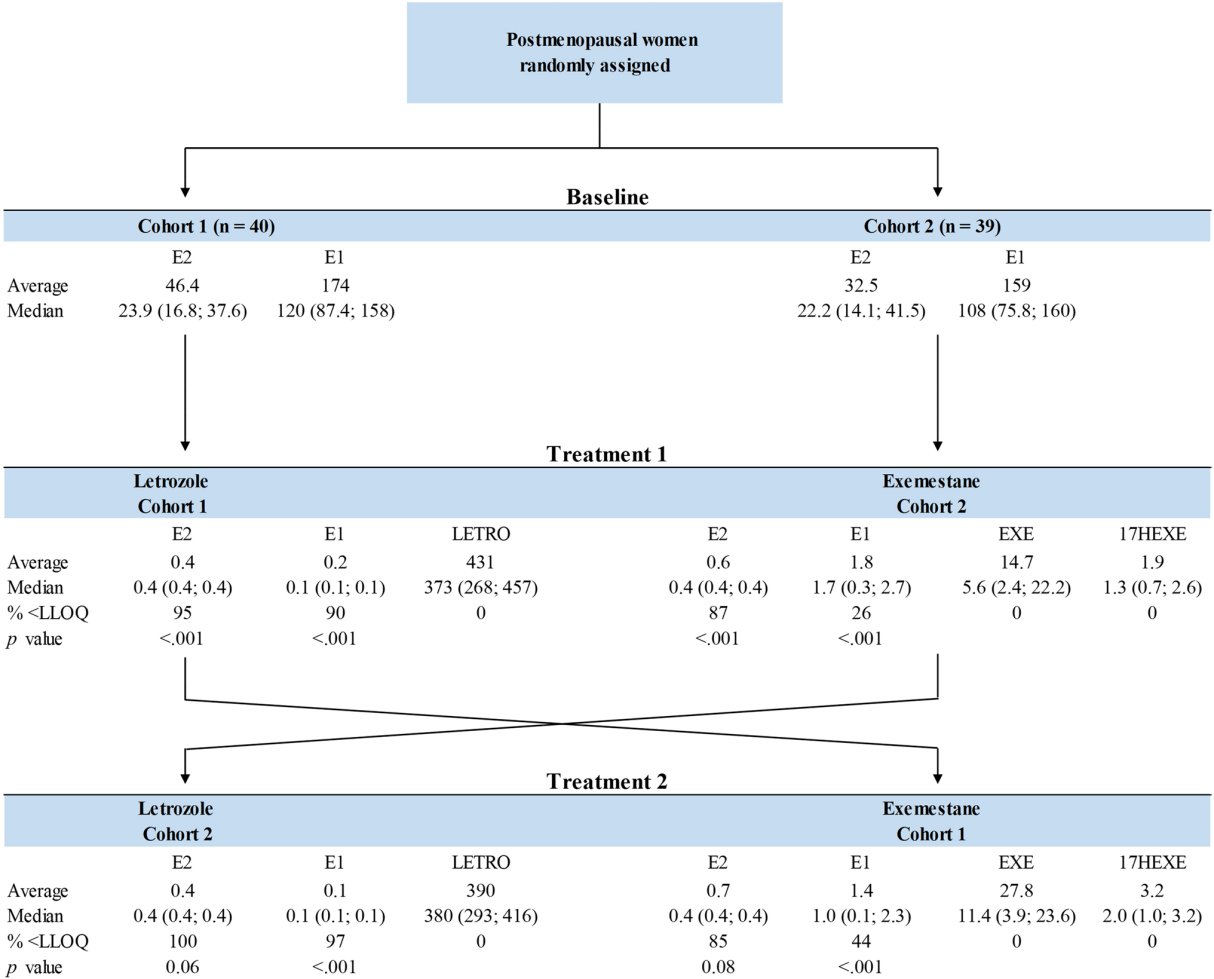
Statistical overview of serum measurements. The figure presents mean values, ranges, and medians for serum levels of Estradiol (E2), Estrone (E1), Exemestane (EXE), 17-hydroxy-exemestane (17HEXE), and Letrozole (LET). P values are calculated comparing each treatment phase to its preceding period (i.e., baseline to treatment 1 and treatment 1 to treatment 2) within each cohort

**Fig. 3:**
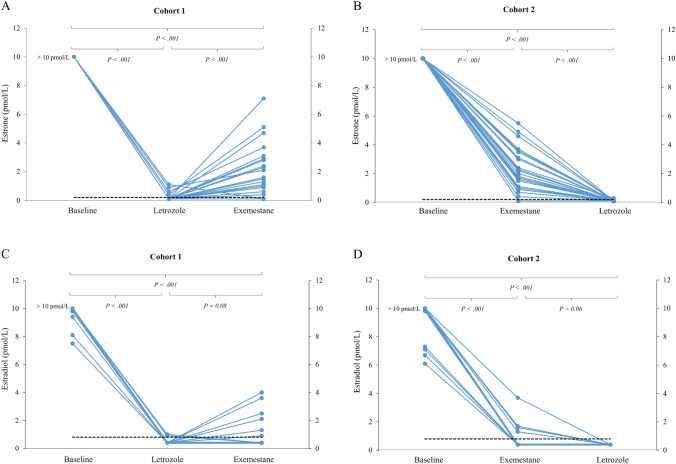
Individual changes in serum estrogen concentrations across treatment periods. Variations in serum levels of estrone and estradiol for individual patients in both cohorts during treatment with either letrozole or exemestane are depicted. P values are calculated comparing each treatment phase to its preceding phase (i.e., baseline to treatment 1 and treatment 1 to treatment 2) within each cohort. The black dashed line represents the lower limit of quantification divided by 2, specifically 0.2 pmol/L for estrone and 0.8 pmol/L for estradiol

*Patient cohort 1*. At baseline, the mean serum estrone and estradiol levels in cohort 1 were 174 pmol/L and 46.4 pmol/L, respectively (Fig. 2). Treatment with letrozole suppressed serum estrone and estradiol levels to a mean value 0.2 pmol/L and 0.4 pmol/L, respectively (*P* < 0.001). It is important to mention that 95% of all samples in this cohort had estradiol levels below the LLOQ during letrozole exposure. When crossed over to exemestane as the second therapy, mean serum levels of estrone and estradiol increased to 1.4 pmol/L and 0.7 pmol/L, respectively. The changes in estrogen concentrations for individual patients are given in Fig. 3.

**Fig. 4:**
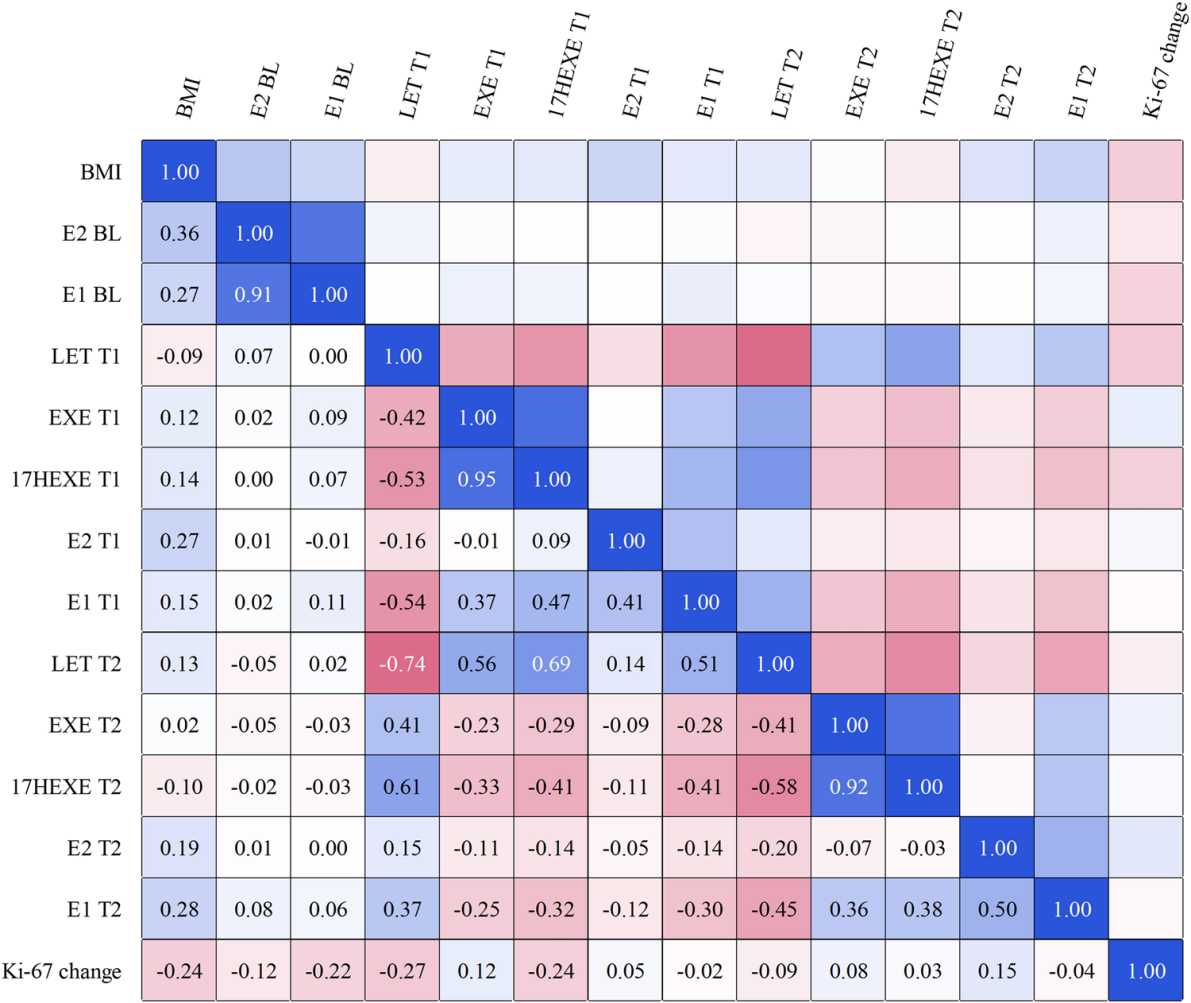
Correlation matrix of key parameters. The matrix displays the correlations between BMI, baseline and treatment-specific levels of estradiol (E2), estrone (E1), letrozole (LET), exemestane (EXE), 17-hydroxy-exemestane (17HEXE), and changes in Ki-67 across the two treatment periods (T1 and T2). Correlations are calculated using Pearson’s r

*Patient cohort 2*. Mean serum levels of estrone and estradiol at baseline were 159 pmol/L and 32.5 pmol/L, respectively. During the initial treatment with exemestane, these levels decreased to 1.8 pmol/L for estrone and 0.6 pmol/L for estradiol (*P* < 0.001). Following cross-over to letrozole, mean serum levels of estrone and estradiol were further suppressed to 0.1 pmol/L for estrone and 0.40 pmol/L for estradiol, respectively (Fig. 2). All relevant correlations between estrogen levels and other factors, such as drug levels, are summarized in Fig. 4.

### Serum drug levels of letrozole and exemestane during neoadjuvant therapy.

**Fig. 5:**
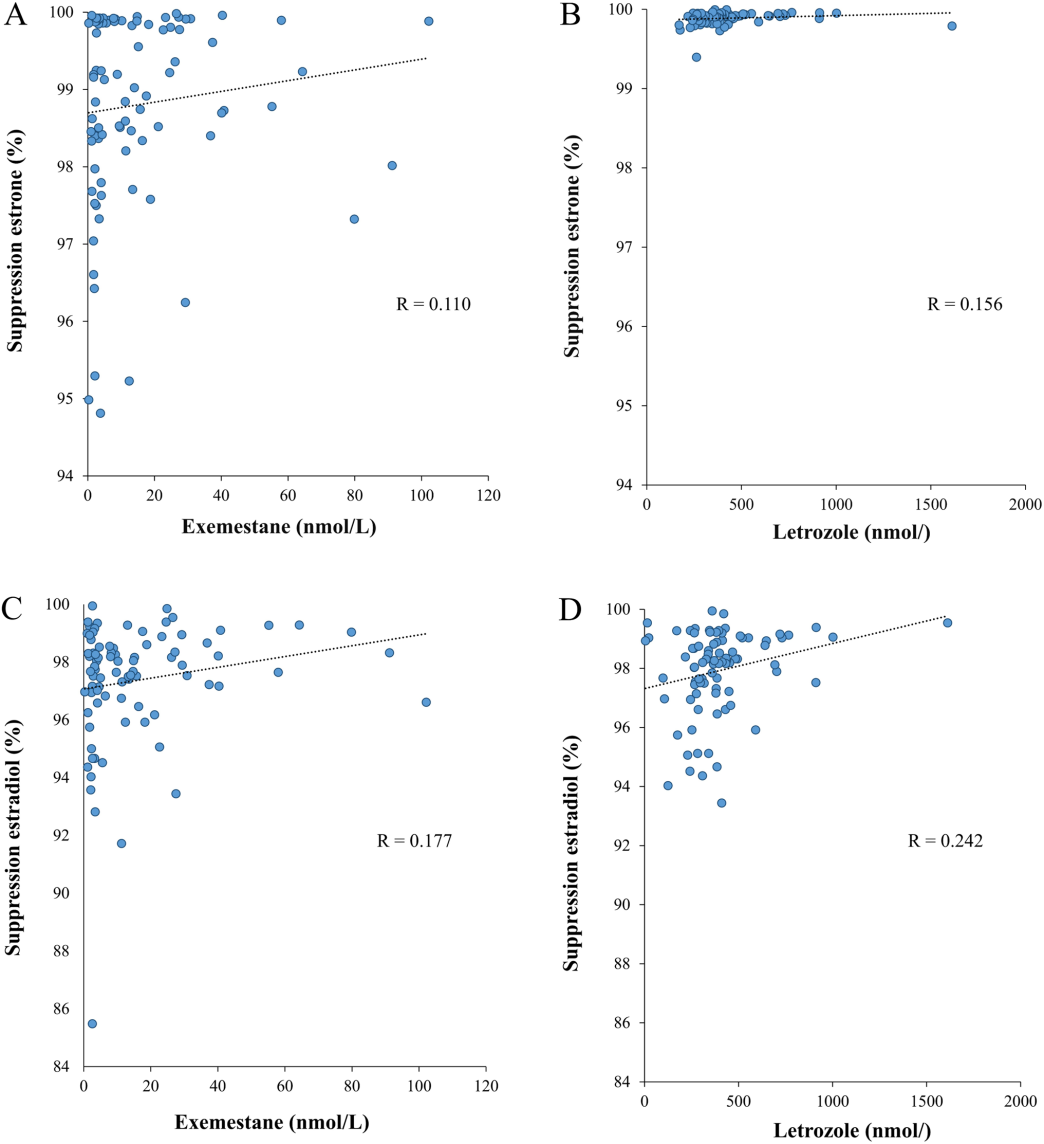
Relationship between serum drug concentrations and estrogen suppression. Graphs A and B illustrate individual patient estrone suppression from baseline to on-treatment with exemestane (graph A) or letrozole (graph B). Graphs C and D present the corresponding data for estradiol suppression. Estrone suppression is more pronounced during letrozole treatment (graph B) compared to exemestane (graph A)

**Fig. 6:**
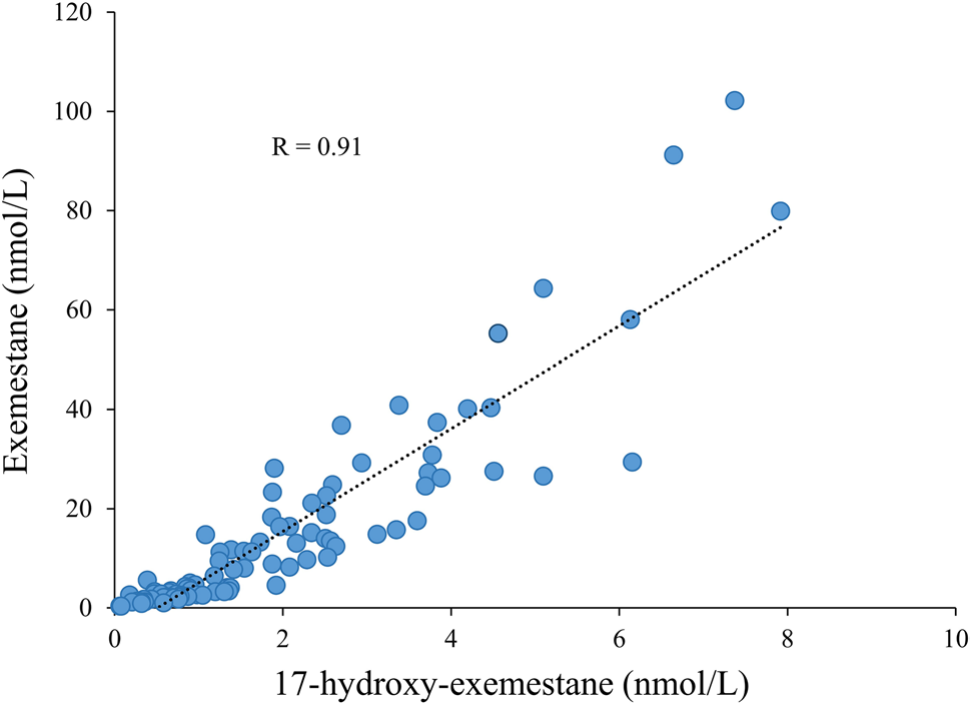
Correlation of serum levels of exemestane and its main active metabolite, 17-hydroxy-exemestane, across individual patients

The measurement of serum levels of letrozole and exemestane confirmed that all patients had adhered to the recommended drug regimen. Specifically, serum drug measurements for individual patients revealed therapeutic levels of both letrozole and exemestane throughout the treatment period. Furthermore, suppression of serum estrone and estradiol levels were clearly correlated to serum drug concentrations, as summarized in Fig. 5. Interestingly, the suppression of serum estrone was more uniform during treatment with letrozole (Fig. 5B) compared to exemestane (Fig. 5A). When examining individual patient data, we observed that suppression of estrone during exemestane ranged from 95 to 100%, in contrast to the picture seen with letrozole, where all patients experienced over 99% suppression (Fig. 5B). The pharmacological important conversion of exemestane to its main active metabolite, 17-hydroxy-exemestane, is documented and summarized for all individual patients in Fig. 6.

### Ki-67

**Fig. 7:**
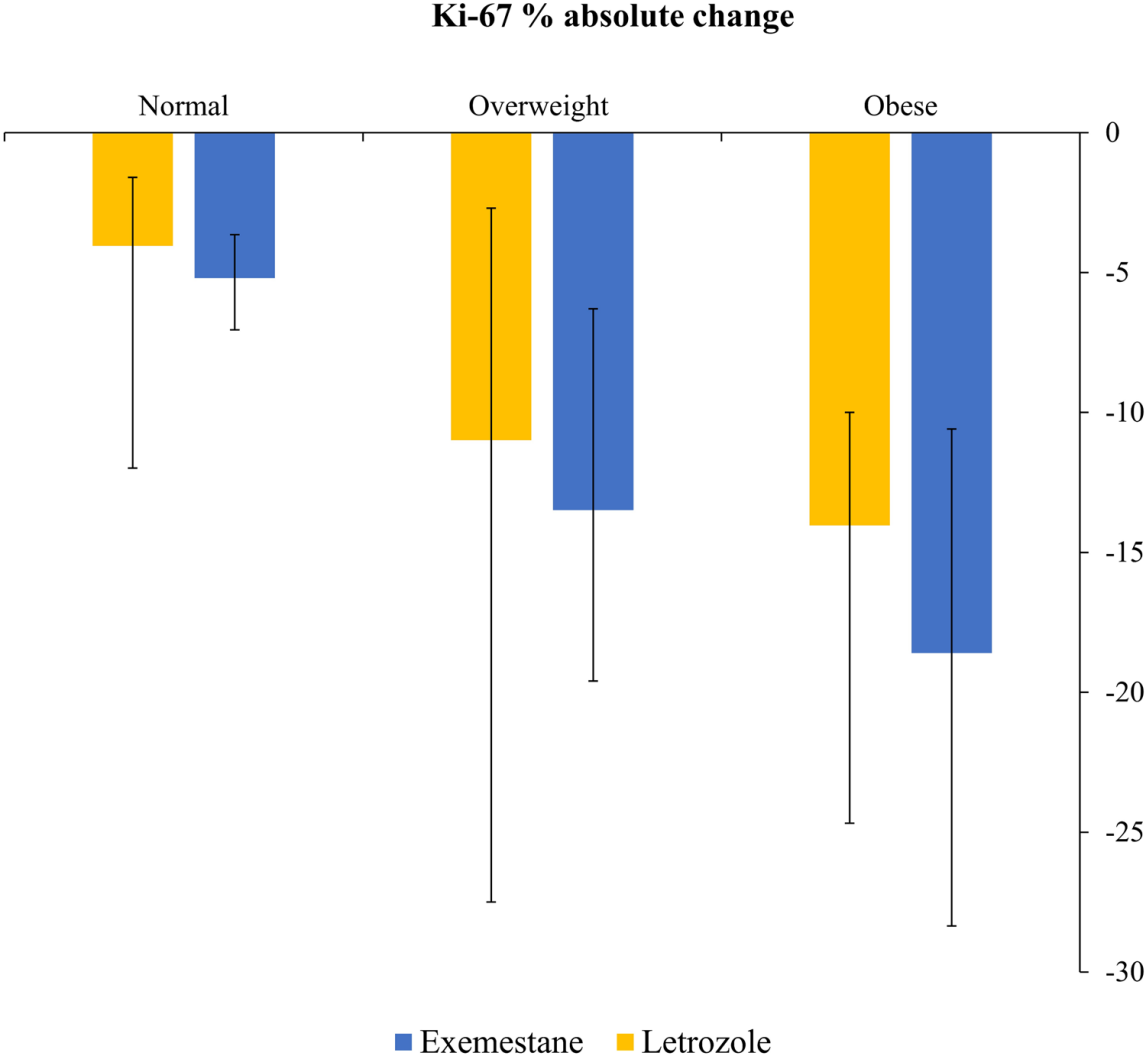
Relationship between BMI and Ki-67 suppression across treatment groups. The figure illustrates the absolute changes in Ki-67 levels in relation to patient BMI categories, stratified by treatment with either letrozole or exemestane. Median changes in Ki-67 (Q2) are represented for each BMI category within both cohorts and reflect measurements from baseline to the conclusion of the 6-month treatment period. Error bars indicate the interquartile range, extending from the first quartile (Q1) to the third quartile (Q3)

Immunohistochemistry (IHC) data for Ki-67 were available for most patients at both baseline and time of surgery. Overall, Ki-67 levels dropped by 68% in cohort 1 and by 66% in cohort 2, when measured from baseline to the levels observed after 6 months on treatment. In women with a normal BMI (18–25), Ki-67 levels decreased by 49 and 63% compared to baseline, when letrozole or exemestane were given during the last 3 months prior to surgery. The decrease in Ki-67 levels from baseline to surgery was 64% with letrozole and 65% with exemestane in women characterized with a BMI between 25 and 30 (overweight). In obese women (BMI 30–39), the relative reduction in Ki-67 levels was 84% during letrozole treatment and 81% during exemestane treatment. The absolute changes in Ki-67 levels during treatment with letrozole and exemestane in correlation to the patient´s BMI are summarized in Fig. 7.

## Discussion

Following limited clinical success of the aromatase inhibitors belonging to the first and second generation, the third-generation drugs (letrozole, anastrozole, exemestane) established this elegant endocrine intervention as the gold standard in postmenopausal women suffering from ER-positive breast cancer [1]. From the early days, aromatase inhibitors were selected for further drug development mainly based on their ability to suppress estrogen levels in vivo. However, due to the limited availability of reliable laboratory methods to estimate tumor tissue estrogen levels, blood estrogen levels were mostly used as surrogate parameters to evaluate the endocrine effects of AIs in humans [20]. Importantly, many established serum methods developed for routine measurement of estrogen levels in postmenopausal women proved to be of limited value due to their high detection limits, failing to distinguish between normal postmenopausal estrogen levels and the profound suppression achieved by the extremely potent drugs of the third generation of AIs. Thus, we and others developed improved methods to measure estrogen levels in the ultra-low range [13, 21–24].

Previously, our group published a head-to-head comparison between the two non-steroidal AIs and direct competitors, anastrozole and letrozole, concerning their suppression of blood estrogen levels in vivo. Our findings demonstrated a significantly more effective suppression of both estradiol and estrone during letrozole therapy compared to anastrozole [7]. These important findings were subsequently corroborated by others showing again a greater suppression of plasma estrogen levels in postmenopausal women with early breast cancer during letrozole therapy as compared to anastrozole [25, 26]. In conclusion, letrozole is currently the most potent non-steroidal, reversible aromatase inhibitor for suppressing free estrogens in the bloodstream when administered at the established daily dose of 2.5 mg.

In contrast, plasma estrogen levels during therapy with the steroidal aromatase inactivator exemestane are significantly more challenging to measure as the steroidal compound exemestane, given at a daily dose of 25 mg, might interfere with the steroid measurements. Nevertheless, we were previously able to measure plasma estrogen levels also during therapy with exemestane and revealed a substantial suppression of both estrone and estradiol using a high pressure liquid chromatography (HPLC) radioimmunoassay (RIA) [6]. However, due to the high costs of radioactive waste disposal, difficulties in the automation of RIAs and lengthy counting times, novel methods have been developed to address these issues.

The current study employed a novel, state-of-the-art LC–MS/MS method with detection limits in the sub-picomolar range [13]. An additional advantage of this method is the ability to measure serum drug levels of all third-generation AIs concurrently, thereby facilitating a compliance check, making this method particular useful for the handling of blood samples collected during AI therapy. In our study, all patients had in fact pharmacological meaningful drug levels at all time points, likely due to the severe clinical situation of the participating patients.

In a neoadjuvant setting, employing an intra-patient cross-over designed study and using the clinically established drug doses, we confirmed letrozole as the superior suppressor of both serum estrone and estradiol levels when compared directly to exemestane. To the best of our knowledge, this is the first direct head-to-head comparison in an intra-patient cross-over designed study of these two pivotal AIs, which continue to represent the two most potent drugs belonging to both pharmacological categories. However, it is important to underline that our findings presented here are purely covering the suppression of estrogen levels in human blood samples during letrozole and exemestane therapy and not the clinical efficacy of different AIs per se. Given the large inter-patient variation concerning estrogen production and metabolism, this study design is considered to be superior to more conventional trials using different patient populations randomized to different aromatase inhibitors [9].

Our results presented here align closely to the data observed when using an alternative method to measure the “total estrogen activity” in human blood samples from the same clinical trial. Thus, we have shown previously a significant better suppression of the “total estrogen activity” in human blood samples during letrozole therapy compared to exemestane using the AroER tri-screen assay, established in the laboratory of Prof. Shiuan Chen at the City of Hope National Cancer Center, Duarte, California [27, 28].

Beyond identifying the most potent suppressor of estrogen levels in vivo, our findings are also important in the context of the clinical observed incomplete cross-resistance between letrozole and exemestane in metastatic breast cancer (MBC). Several clinical trials have demonstrated that exemestane monotherapy can yield clinical beneficial effects in patients with ER-positive MBC when progressing on a non-steroidal AI like letrozole or anastrozole [14, 15]. This observation is paradoxical, given the inferiority of exemestane as a suppressor of estrogen levels compared to letrozole. Consequently, it raises the possibility that exemestane may exert additional antitumor effects that are not solely related to estrogen manipulation, thereby explaining the clinical observation of multiple tumor responses when non-steroidal AIs and exemestane are given in sequence.

In this context, it should be mentioned that we have previously shown that a potent suppression of serum leptin levels could be observed exclusively during exemestane treatment, but not during letrozole therapy. This may partially explain the demonstrated incomplete cross-resistance between exemestane and other AIs [29]. Whether this additional effect of exemestane on a pivotal adipocytokine like leptin is the entire explanation of the antitumor effects of exemestane when given to patients following progression on a non-steroidal AI in MBC is currently unknown. At this point, it cannot be ruled out that additional unknown effects of exemestane, unrelated to its nature as an AI, may contribute. For instance, androgenic effects, potentially involving the androgen receptor, may be involved as well [30]. Additional investigations by our group and others are currently ongoing to further clarify potentially beneficial androgenic effects during treatment with exemestane in BC patients.

While both drugs significantly suppressed Ki-67 levels, irrespectively of their sequence of administration, an intriguing pattern emerged when we examined the data in relation to the patients BMI. Thus, women with a normal BMI (18–25) experienced a relative decrease in Ki67 levels by 49 and 63% during therapy with letrozole or exemestane, respectively. In contrast, in obese women, Ki-67 levels decreased by 84 and 81% during letrozole and exemestane therapy, respectively. While the underlying mechanisms for this disparity remain speculative, it is plausible that the elevated estrogen production in obese women may allow a more substantial drop in estrogen levels during aromatase inhibitor therapy, thereby resulting in greater suppression of Ki-67 levels as well.

While this publication is highly focusing on the suppression of serum estrogens, it should be mentioned that tissue estrogen measurements have been performed during therapy with aromatase inhibitors as well, although less extensive, and not necessarily mirroring the findings made in plasma samples [8]. Thus, it is the general opinion that plasma estrogen levels do not correlate well with breast cancer tissue estrogen levels in general [31]. The intratumoral estrogen disposition in breast cancer is probably predominantly dependent on uptake of estrogens from the circulation and binding to the ER [32], while additional factors like the intra-tumor aromatase activity may contribute to the final concentration of intra-tumor estrogens in ER-positive breast cancer as well [33] among other factors.

Finally, it is important to underline that our findings presented here do not impact on the current clinical use of AIs as pivotal, large clinical trials have shown no significant differences in the efficacy comparing anastrozole and letrozole (NCIC CTG MA.27-trial/FACE-trial), as well as anastrozole, letrozole, and exemestane in early breast cancer patients (FATA-GIM3-trial) [34–36].

In conclusion and to the best of our knowledge, this is the first direct head-to-head intra-patient cross-over comparison of the two pivotal CYP19/aromatase disrupting drugs, letrozole and exemestane, concerning serum estrogen suppression using an intra-patient cross-over designed study. Utilizing a state-of-the-art LC–MS-MS assay, our data clearly demonstrate a superior suppression of both serum estrone and estradiol levels during treatment with letrozole. Our results further underline that the clinically observed phenomenon of an incomplete cross-resistance between these two drugs cannot be attributed to their abilities to suppress estrogen levels, suggesting the involvement of additional, currently not completely understood, mechanisms.

## Data Availability

All datasets used and/or analyzed during the current study are available from the corresponding author on reasonable request.
